# Correlation between Radiological Images and Histopathological Type of Meningioma: A Cohort Study

**DOI:** 10.4314/ejhs.v32i3.16

**Published:** 2022-05

**Authors:** Andi Ihwan, Rauf Rafika, Muhammad Husni Cangara, Kevin Jonathan Sjukur, Muhammad Faruk

**Affiliations:** 1 Department of Neurosurgery, Faculty of Medicine, Hasanuddin University, Makassar, Indonesia; 2 Department of Neurosurgery, Wahidin Sudirohusodo Hospital, Makassar, Indonesia; 3 Department of Radiology, Faculty of Medicine, Hasanuddin University, Makassar, Indonesia; 4 Department of Pathology Anatomy, Faculty of Medicine, Hasanuddin University, Makassar, Indonesia; 5 Department of Surgery, Faculty of Medicine, Hasanuddin University, Makassar, Indonesia

**Keywords:** Meningioma, Pathology, Diagnosis, Computed Tomography, Imaging

## Abstract

**Background:**

Histologically affirmed meningiomas represent 37.6% of all essential central nervous system tumors and half of all types of critical central nervous system tumors. This study compares computed tomography (CT) scans of the head with histological findings to establish the characteristics of different types of meningiomas observed in eastern Indonesia.

**Methods:**

This prospective study evaluated 224 patients by examining the correlation between histological and CT data collected from January to December 2020 at Wahidin Sudirohusodo Hospital, Makassar, Indonesia. We assessed data including the location of pre- and post-contrast CT scans, number of tumors, margin, density, contrast enhancement, bony reaction, calcification, and perifocal edema. Patients underwent biopsies followed by an examination of the anatomical pathology tissue.

**Results:**

The female-to-male ratio of participants was 4.2 to 1, and the highest incidence was observed in participants of both genders aged 40–60 years. The most common meningioma subtype was meningothelial, while the most commonly observed locations involved the convexity and sphenoid regions. Most meningiomas had well-defined margins on CT imaging: 54.5% of patients exhibited isodense lesions on pre-contrast scans, and 64.7% exhibited high-contrast enhancement. Bone destruction developed in 4.1% of patients, while hyperostosis was observed in 17.4%, and calcification was present in 10.3% of the participants. Edema was identified in 65.2% of cases, of which moderate edema was the most common manifestation.

**Conclusion:**

Meningioma should be highly suspected in female patients aged 40–60 with isodense lesions on pre-contrast CT scans and high-contrast enhancement on post-contrast CT scans. Meningiomas were primarily classified as convexity meningiomas with well-defined margins. The presence of hyperostosis, calcification, and brain edema supported the meningioma diagnosis.

## Introduction

Meningioma is the best-known non-glial essential tumor of the central nervous system and the most widely recognized extra-axial neoplasm emerging from the arachnoid cap cells. In 2015, a systematic review estimated the worldwide annual incidence of essential cerebrum tumors at 10.82 per 100,000 individuals (1). Between 2010 and 2014, the annual incidence was 8.3 per 100,000 people, suggesting that the incidence is increasing (2). The mean age of presentation was 66 years, with a female-to-male ratio of 2.3 to 1 (3–5). A higher rate was observed in the African American population, in which the female-tomale ratio was similar, at 2.3 to 1 (2, 3).

Histologically affirmed meningiomas represented 37.6% of all essential central nervous system tumors and half of all types of essential central nervous system tumors. This condition affects approximately 1.8 to 13 out of 100,000 people annually (6). The prevalence in the United States is 97.5 per 100,000 people, with more than 170,000 individuals diagnosed with meningiomas (7). In Indonesia, 13–26% of intracranial tumors have been diagnosed as meningiomas (8). In terms of age, meningiomas are more common in adults than in children, with an incidence of 37.75 per 100,000 individuals in people aged 75 to 84 years compared to 0.14 per 100,000 in children aged 0 to 19 years (2).

Two types of risk factors, intrinsic and extrinsic, contribute to meningioma. Intrinsic risk factors for meningioma include age, sex, ethnic group, and family history; genetic polymorphisms of Turner syndrome, Werner syndrome, and neurofibromatosis type 2; and family cancer syndromes, including neurofibromatosis type I (NF1), patched mutations (PTCH), CREB-binding protein (CREBBP), Von Hippel-Lindau (VHL), Phosphatase and Tensin Homolog deletion (PTEN), and the cyclin-dependent kinase inhibitor 2A (CDKN2A) genes. Extrinsic risk factors include electromagnetic fields; previous head injury; and nutritional, toxic, and hormonal factors (9).

Typical computed tomography (CT) features on unenhanced images included a well-defined, smoothly marginated extra-axial mass adjoining the dura mater (10). Non-contrast CT imaging meningiomas revealed that 60% of lesions were slightly hyperdense compared to normal brain tissue, while the remaining lesions were more isodense. Post-contrast CT imaging revealed that 72% of lesions were brightly and homogeneously contrast-enhanced (11). Meningioma calcification occurred in a spotted, rim-like, or nodular pattern and was observed in 20–30% of patients (12). Over half of meningiomas exhibited a variable amount of vasogenic edema in the adjacent brain parenchyma (13). Hyperostosis was the most common related bony finding; this condition appeared as a bony thickening on CT and was observed in 25–49% of meningiomas, of which convexity and sphenoid wing meningiomas were the most common (14).

Imaging plays a crucial role in identifying meningioma and facilitating the differentiation of pre-surgical findings, an essential part of improving treatment strategies (15). This study discusses the correlation between the anatomical pathology of meningioma and advanced imaging features, using CT head scans to define the characteristics of the different types of meningiomas observed in eastern Indonesia based on their histology.

## Materials and Methods

A descriptive study with prospective analysis (cohort study) was conducted using data from 224 patients with meningioma (ICD-10 codes D-32: meningioma, benign; D-42: meningiomatosis, uncertain whether benign or malignant; C-70: meningioma, malignant, primary site) involving medical records at Wahidin Sudirohusodo Hospital, Makassar, Indonesia. The data were evaluated by histological correlation in patients aged 21–80 years from January to December 2020. The study was registered under research registry no. 7127 and reported according to the *Strengthening the Reporting of Cohort Studies in Surgery* (STROCSS) guidelines (16, 17).

Meningioma was diagnosed by physical examination, CT imaging, and examination of the anatomical pathology tissue. Patients aged 21 years or older who agreed to a biopsy were included in the study. The exclusion criteria were recurrent meningiomas and primary tumors in other locations or metastases.

CT scans were carried out using a Somatom go. Top 128 Slice Scanner machine (Siemens, China) using the standard CT protocol for the head and neck. Non-ionic contrast media were routinely administered in all patients to examine the enhancement pattern and characteristics. Patients with suspected meningioma were evaluated with pre- and post-contrast CT imaging. After these sequences were obtained, an intravenous contrast study was performed for all patients to assess the degree and pattern of enhancement as well as vascularity. We evaluated the following CT data: location, number of tumors, margin, density, contrast enhancement, bony reaction, calcification, and perifocal edema. Moreover, perifocal edema on CT scans was divided into 3 categories: mild (<1 cm thickness), moderate (1–2 cm thickness), and severe (>2 cm thickness).

Meningiomas could be hypodense, isodense, or hyperdense on the observed CT scans. A range of non-contrast enhancement to high-contrast enhancement was evident on post-contrast CT scans. Solid or multiple meningiomas were also marked with well-defined, indistinct, or irregular margins. Edema, calcification, and bony reaction were also present in some cases. The location of intracranial meningiomas could be found in the convexity, parasagittal, sphenoid wing, infratentorial, interventricular, tuberculum *sellae*, and other sites (10, 11, 14, 18, 19).

Patients whose head scans suggested meningiomas were asked to provide consent for a biopsy. Patients who agreed to undergo a biopsy received a biopsy, followed by examining the anatomical pathology tissue. The 2016 WHO classification system, which is based on the pathological evaluation of meningioma tissue, includes 15 subtypes divided into 3 categories according to cell type (20). Type I include meningothelial, fibroblastic, transitional, angiomatous, psammomatous, microcystic, secretory, lymphoplasmacytic-rich, and metaplastic; type II are chordoid, clear cell, and atypical; and type III comprises papillary, rhabdoid, and anaplastic (9, 21).

**Ethical Approval**: The ethical approval of this study was granted from Ethical Committee, Faculty of Medicine, Hasanuddin University Makassar, Indonesia.

## Results

Patients ranged from 21 to >70 years old, most of whom were between age 40 and 60. The majority of patients with meningioma were female (80.8%); thus, 19.2% were male. Most of the lesions (n = 54, 24.1%) were located in the frontal region. We found some (n = 48, 21.4%) in the parietal and temporal regions, and 42 (18.8%) were located in the sphenoid. Lesions were less common in the falx cerebri, occipital, suprasellar, retrobulbar, and posterior cranial fossa regions (Table 1).

**Table 1:** Patient characteristics

Age (years)	Number of cases	Percent
21–30	4	1.8
31–40	18	8
41–50	74	40.3
51–60	78	34.8
60–70	48	21.4
>70	2	0.9
**Gender**		
Male	43	19.2
Female	181	80.8
**Location**		
Frontal	54	24.1
Parietal	48	21.4
Falx	18	8
Fossa posterior	2	0.9
Occipital	5	2.2
Sphenoid	42	18.8
Temporal	48	21.4
Suprasellar	4	1.8
Retrobulbar	3	1.4

**Figure 1:**
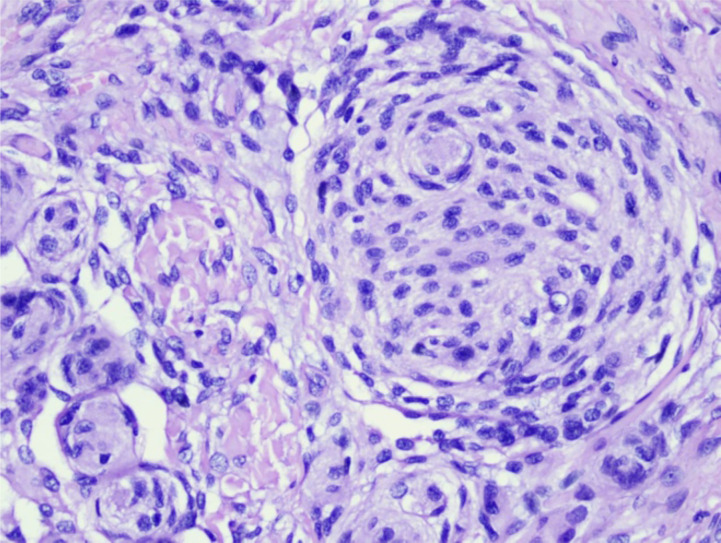
A meningothelial meningioma, classified by histopathology with hematoxylin-eosin staining, magnification 40×. The following characteristics were noted: proliferation of meningothelial cells with round nuclei, relatively monotonous, not atypical, vesicular chromatin, and non-prominent nucleoli

Almost all patients (n = 201, 89.7%) had one lesion. More than half of the patients (n = 117, 52.2%) had meningothelial histopathological subtypes (Figure 1) featuring a single dominant lesion (94.7%).

Some (n = 24, 10.8%) were atypical meningioma, 22 (9.8%) were fibroblastic meningiomas, 18 (8%) were anaplastic meningiomas, and 15 (6.8%) were transitional meningiomas. Less common meningioma histopathological subtypes were clear cell, angiomatous, psammomatous, choroid, and papillary meningioma. Multiple meningiomas were observed in 23 patients (10.3%); these comprised angiomatous meningioma (42.9%), anaplastic meningioma (22.2%), atypical meningioma (16.7%), fibroblastic meningioma (13.6%), and meningothelial meningioma (7.7%), as shown in Table 2.

**Table 2:** Characteristics of meningiomas on CT imaging based on their margin

Meningioma histopathological subtype	Total cases	Number of tumors		Margin		

		Single	Multiple	Well-defined	Indistinct	Irregular
Meningothelial	117	108	9	114	3	0
Fibroblastic	22	19	3	20	2	0
Transitional	15	15	0	15	0	0
Angiomatous	7	4	3	7	0	0
Psammomatous	5	5	0	5	0	0
Chordoid	4	4	0	4	0	0
Clear cell	8	8	0	8	0	0
Atypical	24	20	4	22	2	0
Papillary	4	4	0	4	0	0
Anaplastic	18	14	4	12	0	6

Of the 224 patients, 211 (94.2%) had well-defined margins on CT imaging. Meanwhile, indistinct margins were observed in 7 patients (3.1%); these were meningothelial (2.6%), fibroblastic (9.1%), and atypical meningiomas (8.3%). Irregular meningioma margins were observed in 6 patients (2.7%); these meningiomas were classified as anaplastic, as shown in Table 2.

**Figure 2:**
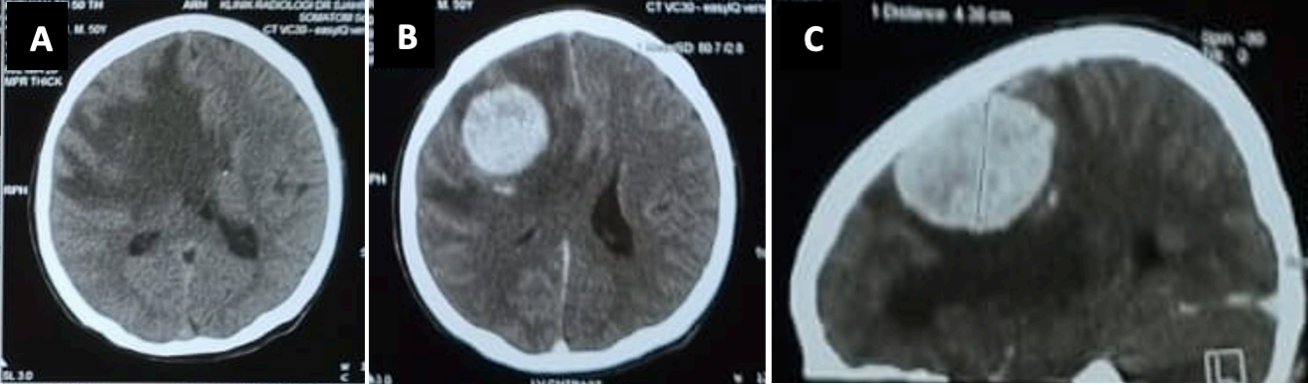
Male, 50 years old, with cephalgia, seizure, and left hemiparesis. An isodense lesion was observed in the right frontotemporal on an axial plane pre-contrast CT scan (A). A contrast enhancement lesion and severe perifocal edema were observed on the axial and sagittal plane post-contrast CT scan (B and C).

Of the 224 patients who had pre-contrast CT imaging, 122 (54.5%) exhibited isodense lesions (Figure 2A), while 60 patients (26.8%) exhibited hyperdense lesions, and 42 patients (18.7%) exhibited hypodense lesions. Isodense lesions were dominant in almost all meningioma subtypes, except for choroid meningiomas (of which 2 [50%] were isodense, and 2 [50%] were hypodense) and psammomatous meningiomas (of which 3 [60%] were primarily hyperdense). Isodense lesions were observed in 62 cases of meningothelial meningioma (53%), 12 cases of fibroblastic meningioma (54.5%), 7 cases of transitional meningioma (46.7%), 3 cases of angiomatous meningioma (42.9%), 5 cases of clear cell meningioma (62.5%), 15 cases of atypical meningioma (62.5%), 3 cases of papillary meningioma (75%), and 11 cases of anaplastic meningioma (61.1%) (Table 3).

**Table 3:** Density of meningioma on CT imaging

Meningioma histopathological subtype	Total cases	Pre-contrast imaging			Post-contrast imaging		

		Hypodense	Isodense	Hyperdense	Non-contrast enhancement	Minimal contrast enhancement	High contrast enhancement
Meningothelial	117	23	62	32	5	38	74
Fibroblastic	22	5	12	5	0	7	15
Transitional	15	3	7	5	1	6	8
Angiomatous	7	2	3	2	0	1	6
Psammomatous	5	0	2	3	0	0	5
Chordoid	4	2	2	0	0	1	3
Clear cell	8	0	5	3	0	1	7
Atypical	24	2	15	7	3	6	15
Papillary	4	0	3	1	0	2	2
Anaplastic	18	5	11	2	2	6	10

More than half of the meningioma cases (N = 145, 64.7%) featured high-contrast enhancement (Figure 2B and 2C), excluding papillary meningioma, which had an equal proportion of minimal and high-contrast enhancement: 5 cases of psammomatous meningioma (100%), 7 (87.5%) cases of clear cell meningioma, 6 cases of angiomatous meningioma (85.7%), 3 cases of chordoid meningioma (75%), 15 cases of fibroblastic meningioma (68.2%), 38 cases of meningothelial meningioma (63.2%), 15 cases of atypical meningioma (62.5%), 10 cases of anaplastic meningioma (55.5%), and 8 cases of transitional meningioma (53.3%) (Table 3).

Of the 224 participants, 9 (4%) developed bone destruction, 39 (17.4%) had hyperostosis (Figure 4), and 78 (34.8%) did not develop any bone defects. Twenty-three patients (10.3%) had calcification, which was found in 18 cases of meningothelial meningioma (78.3%), 2 cases of fibroblastic meningioma (8.7%), 1 case of transitional meningioma (4.3%), 1 case of psammomatous meningioma (4.3%), and 1 case of anaplastic meningioma (4.3%). Edema was found in 146 cases (65.2%); moderate brain edema was observed in 109 patients (74.7%), whereas mild and severe brain edema was observed in 26 (17.8%) and 11 (7.5%) patients, respectively. Seventy-eight patients (34.8%) did not have edema, as shown in Table 4.

**Table 4:** CT findings in meningioma

Meningioma histopathological subtype	Total cases	Bone affection		Calcification	Edema		
	
		Hyperostosis	Bone destruction		Mild	Moderate	Severe
Meningothelial	117	27	7	18	14	81	9
Fibroblastic	22	3	0	2	4	7	0
Transitional	15	1	0	1	1	3	1
Angiomatous	7	0	0	0	1	1	0
Psammomatous	5	0	0	1	0	2	0
Chordoid	4	0	0	0	0	0	0
Clear cell	8	0	0	0	0	1	0
Atypical	24	1	1	0	1	9	1
Papillary	4	0	0	0	0	0	0
Anaplastic	18	7	1	1	5	5	0

## Discussion

The female-to-male ratio in our study was 4.2 to 1. This proportion is equivalent to a study by Ostrom et al., who reported more meningioma cases in women than in men (3). However, whereas Buerki et al. reported peak incidence in the 75-to-84 age group, meningiomas in our cohort most often occurred in patients aged between 41 to 60 years (67.8% of patients).

According to the WHO grades for meningioma, the most prevalent meningioma subtypes in our institution were meningothelial meningioma, followed by the atypical and fibroblast meningiomas. Meningothelial and fibroblast meningioma were grade I meningiomas, accounting for 74% of all meningiomas observed. Clear cell meningioma, chordoid meningioma, and atypical meningioma represented sixteen percent. Ten percent involved anaplastic meningioma and papillary meningioma. Ostrom et al. (22) found 81.1% are considered typical or grade I, and 16.9% are considered atypical or grade II, similar to the results of our study. The prevalence of anaplastic or grade III meningiomas was 1.7%, lower than the prevalence observed in our study. A study by Mary et al. (10) in 2005 at Kenyatta National Hospital (KNH) found grade I tumors to be the most common, accounting for 80.1% of meningiomas. Meanwhile, grade II and III tumors represented 15.9% and 4% meningiomas, respectively.

The most common subtypes identified by Mary et al. (10) at KNH were meningothelial and transitional, totaling 35% and 30% of meningiomas, respectively. In contrast, Wanjeri identified fibroblastic, transitional, and meningothelial in 25.4%, 25.4%, and 22.5% of meningiomas, respectively. However, our study found meningothelial meningiomas characterizing 52.2% of the observed lesion. Sheporaitis et al. (23) reported that meningiomas are most often solitary, with one series demonstrating multiple meningiomas on CT in 8.9% of patients. Similarly, almost all patients in our study (89.7%) had one lesion.

Typical diagnostic highlights were found on CT in 72–85% of cases, including a well-defined lobe mass with a broad-based dural attachment (14). In our study, most of the patients had well-defined margins on CT imaging.

Nasrin et al. (20) reported the distribution of intracranial meningiomas in most instances as follows: cerebral convexity (35%), parasagittal (20%), sphenoid wing (20%), infra-tentorial (13%), interventricular (5%), tuberculum sellae (3%), and other sites (4%). In our study, the most common location of meningiomas was also the convexity, accounting for 69.2% of meningiomas; of these, 24.1% were located in the frontal region. The parietal and temporal region (21.4%) was the second most common locations.

According to Lyndon et al., (24) pre-contrast CT imaging revealed 60% of lesions to be slightly hyperdense compared with normal cerebral cortex tissue, with the remainder being more isodense. However, in our study, more than 50% of patients exhibited isodense lesions on pre-contrast CT imaging, while hyperdense and hypodense lesions were 26.8% and 18,7%, respectively. In Lyndon et al.'s (24) report, post-contrast CT imaging revealed that 72% of lesions were brightly and homogeneously contrast-enhanced; in comparison, 64.7% of the meningioma cases in our study had high-contrast enhancement, excluding papillary meningioma, which exhibited an equal proportion of minimal and high-contrast enhancement.

Hyperostosis, the most common related bony finding, appears as a bony thickening on CT and is observed in up to 25–49% of meningiomas. Gangadhar et al. observed bone destruction to be an uncommon feature, only found in approximately 3% of cases (15). In comparison, our study showed that 17.4% had hyperostosis, while only 4% developed bone destruction.

Calcification was uncommon featobserved in only 10.3% of the study participants, a much lower figure than the 20–30% reported by Greenberg et al. (11). Almost all the calcified meningiomas were grade I (95.7%), of which 81.8% were meningothelial. In our study, calcification was observed in 20% of cases of psammomatous meningioma, 15.4% cases of meningothelial meningioma, 9.1% cases of fibroblastic meningioma, 6.7% cases of transitional meningioma, and 5.5% cases of anaplastic meningioma. No calcification was observed in angiomatous chordoid, clear cell, atypical, or papillary meningioma.

In Kim et al.'s (13) findings, more than half of the meningiomas studied demonstrated a variable amount of vasogenic edema in the adjacent brain parenchyma. Our study showed similar results, where edema was observed in majority of cases, mostly classified as moderate brain edema.

Our results suggest a lower prevalence of calcification and hyperostosis in meningioma compared to other researchers' results. Therefore, further research based on the criteria we included is recommended. We welcome any constructive input or suggestions for this research. We further hope that this research will provide a valuable reference in the field of neurosurgery.

Many pathologies affecting the dura can mimic meningiomas in CT imaging, such as primary neoplastic processes and inflammatory, infectious, and metastatic disease. Limitations of this study include the lack of high-quality raw data, which mainly manifested as significant heterogeneity of the patient cohorts and imaging data, as well as small sample sizes. The original data's heterogeneity may introduce changes that do not represent underlying biological effects. Small sample sizes can lead to higher statistical error rates and an increased risk of overfitting. The previous literature lacks not only original data but also utilization, as many studies only use a portion of the imaging data. For example, some studies have only used the enhancing sequences rather than all CT scan sequences, or the researchers extracted features from a series of consecutive slices rather than all slices.

Our study suggests that meningioma could be highly suspected when cerebral tumor symptoms are present in female patients aged 40–60 years, with isodense lesions with well-defined margins on pre-contrast head CT scans and high-contrast enhancement on post-contrast CT scans. The presence of hyperostosis, calcification, and edema on head CT scans can support meningioma diagnosis. However, the definitive diagnosis of meningioma should be based on anatomical pathology examination to determine the type of meningioma and subsequent therapy.
